# Effective management of recurrent Doege–Potter syndrome with somatostatin‐analogues: A case report

**DOI:** 10.1002/cnr2.1992

**Published:** 2024-03-05

**Authors:** Felix Schöler, Maximilian Andreas Storz, Ashkan Khavaran, Nicolas Hümmler, Maximilian Frederik Russe, Christoph‐Ferdinand Wielenberg, Katharina Laubner, Jochen Seufert

**Affiliations:** ^1^ Division of Endocrinology and Diabetology, Department of Internal Medicine II, University Hospital of Freiburg, Faculty of Medicine University of Freiburg Freiburg Germany; ^2^ Department of Internal Medicine II, Center for Complementary Medicine, University Hospital of Freiburg, Faculty of Medicine University of Freiburg Freiburg Germany; ^3^ Department of Thoracic Surgery, Medical Center University Hospital of Freiburg Freiburg Germany; ^4^ Department of Radiology, Clinic for Diagnostic and Interventional Radiology, University Hospital of Freiburg, Faculty of Medicine University of Freiburg Freiburg Germany; ^5^ Department of Nuclear Medicine, University Hospital of Freiburg Faculty of Medicine Freiburg Germany

**Keywords:** debulking surgery, Doege–Potter syndrome, insulin‐like growth factor II, Lanreotide, Octreotide, solitary fibrous tumor

## Abstract

**Background:**

Doege–Potter syndrome is defined as paraneoplastic hypoinsulinemic hypoglycemia associated with a benign or malignant solitary fibrous tumor frequently located in pleural, but also extrapleural sites. Hypoglycemia can be attributed to paraneoplastic secretion of “Big‐IGF‐II,” a precursor of Insulin‐like growth factor‐II. This prohormone aberrantly binds to and activates insulin receptors, with consecutive initiation of common insulin actions such as inhibition of gluconeogenesis, activation of glycolysis and stimulation of cellular glucose uptake culminating in recurrent tumor‐induced hypoglycemic episodes. Complete tumor resection or debulking surgery is considered the most promising treatment for DPS.

**Case:**

Here, we report a rare case of a recurrent Doege‐Poter Syndrome with atypical gelatinous tumor lesions of the lung, pleura and pericardial fat tissue in an 87‐year‐old woman. Although previously described as ineffective, we propose that adjuvant treatment with Octreotide in conjunction with intravenous glucose helped to maintain tolerable blood glucose levels before tumor resection. The somatostatin‐analogue Lanreotide was successfully used after tumor debulking surgery (R2‐resection) to maintain adequate blood glucose control.

**Conclusion:**

We conclude that somatostatin‐analogues bear the potential of being effective in conjunction with limited surgical approaches for the treatment of hypoglycemia in recurrent or non‐totally resectable SFT entities underlying DPS.

## 1 INTRODUCTION

In 1930, hypoglycemia associated with non‐pancreatic tumor cells in the mediastinum was first reported independently by Doege^1^ and Potter.^2^ Since the early 2000s, the term “Doege–Potter syndrome” (DPS) has been used in multiple case reports to describe recurrent hypoglycemia caused by intrathoracic solitary fibrous tumor cells.^3^, ^4^, ^5^ This condition is relatively rare and occurs in only 5% of patients with solitary fibrous tumors (SFT).^6^, ^7^ The tumor morphology is solid with well‐defined borders in 80% of malignant and 100% of benign cases. At the time of detection, the tumor‐size is usually larger than 10 cm.^8^

Hypoglycemia in DPS is related to paraneoplastic secretion of “Big‐IGF‐II,” a prohormone of insulin‐like growth factor II (IGF‐II).^6^ IGF‐II exerts classical insulin actions such as inhibition of gluconeogenesis, activation of glycolysis and stimulation of cellular glucose uptake by aberrantly binding to insulin receptors.^9^ Physiologically, IGF‐II‐binding‐proteins‐3/5 (IGBP‐3/5) regulate hormone activity by inactivating free IGF‐II through formation of large protein complexes.^10^ However, protein complexes with Big‐IGF‐II are significantly smaller, resulting in enhanced capillary permeability and bioavailability, which in turn enables increased insulin receptor stimulation.^11^, ^12^ In addition, elevated tumor production of Big‐IGF‐II displaces IGF‐II from IGBP‐3/5 binding sites, subsequently raising concentrations of free active IGF‐II.^13^ Increased glucose turnover due to elevated metabolism of tumor cells further contributes to hypoglycemia.^14^

Computed tomographic imaging and percutaneous biopsy are the most effective diagnostic methods for intrathoracic SFT.^15^ In addition, hypoglycemia suppresses endogenous insulin secretion, which can be detected by low serum C‐peptide levels. In patients with DPS, increased blood levels of Big‐IGF‐II or free active IGF‐II may help to confirm the diagnosis. Immunoblot analysis has proven to be a rapid and sensitive method in clinical practice in order to assess these hormones.^10^, ^16^

Complete resection or tumor debulking of the Big‐IGF‐II producing SFT is the most promising treatment for patients with DPS, particularly in light of the fact that the majority of metabolic alterations due to increased Big‐IGF‐II are fully reversible.^10^, ^12^, ^17^ Adjuvant chemotherapy and radiotherapy might also improve the general outcome.^18^, ^19^ Apart from surgery, glucocorticoids may be considered for long‐term therapy, as they appear to suppress secretion of Big‐IGF‐II and may contribute a protective effect for hypoglycemia by increasing insulin resistance.^3^ Despite variable expression of somatostatin receptors (SSTR) in SFT, Octreotide has so far been reported to be ineffective in treatment of DPS.^20^ Only one case report described a slight reduction in the frequency of hypoglycemic episodes. However, Octreotide had no influence on elevated levels of Big‐IGF‐II or suppressed levels of insulin.^21^

Here, we report the rare case of a DPS in an 87‐year‐old woman, which after initial resection recurred atypically as a gelatinous, liquified tumor unsuitable for R0 resection, mandating non‐surgical treatment alternatives. The advanced age of the patient and her personal treatment preferences rendered other treatment options untenable, as well.

## 2 CASE

**FIGURE 1:**
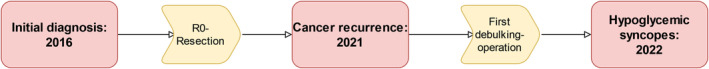
An overview of the patient's course of disease. The patient was first diagnosed with an intrathoracic SFT and DPS in 2016. After initial R0 resection, DPS recurred in 2021. Since complete resection was rendered untenable, a debulking surgery took place. In November 2022 hypoglycemic symptoms reemerged.

We present the case of an 87‐year‐old woman with a history of recurrent intrathoracic SFT, progressive hypoglycemia and known DPS, who was admitted to the University Hospital of Freiburg, Germany in November 2022. DPS was first diagnosed in 2016 and after R0 resection of the SFT, the tumor recurred in 2021 (Figure 1).

Initially the patient was admitted to a tertiary hospital in 2016 with recurrent confusion, palpitations, reduced appetite, halitosis, and hypoglycemia. She denied dyspnea, fever, night sweats, and unintentional weight loss. Her medical history, family medical history and physical examination were unremarkable, except for 30 years of passive nicotine exposure and decreased breath sounds in the left lower thorax.

A CT‐scan showed a mass primarily located in the left thorax. Laboratory chemistry revealed low serum levels of insulin, proinsulin, and C‐peptide. Histology following percutaneous biopsy confirmed the diagnosis of DPS. Tumor extirpation with lower lobe and partial diaphragm resection were performed successfully. Pathological examination confirmed the resection status as R0, with a tumor mass of 11.5 cm × 18 cm × 22 cm, weighing 1300 g. Regular follow‐up thoracic CT‐scans showed a slight pleural effusion, but no further signs of malignancy up to 5 years after the surgery. The patient experienced no further hypoglycemic episodes at this stage.

In 2021, almost 5 years after the initial diagnosis, the patient reported dyspnea (NYHA II), recurring episodes of dizziness, lethargy, and hypoglycemic syncopes. Once again clinical examination showed slightly decreased breath sounds on the left thoracic side. CT‐scans revealed a corresponding tumor mass infiltrating the mediastinum, pericardium, and diaphragm. Due to the tumor morphology, an R0 resection was deemed unfeasible by the treating physicians. Debulking surgery at the same external tertiary hospital revealed multiple gelatinous, liquified tumor lesions instead of a solid tumor at this time.

The successful debulking surgery improved the patient's clinical symptoms, who no longer experienced hypoglycemic episodes. Histological examinations of the resected tumor showed fusiform cells with oval, vesicular, and relatively monomorphic nuclei. The tumor was covered with mesothelium (AE1/3(+)) and was CD34(+), BCL2(+), Vimentin(+), AE1/3(−), CD56(−) with a Ki67 index of 10%. There was no overexpression of p53, one mitosis per high‐power field (HPF) and necrosis in more than 10% of the tumor.

In November 2022, only 1 year after the second surgery, another follow‐up thoracic CT‐ scan detected rapid tumor progression, with a substantial increase in tumor size. Without clinical symptoms for the first 9 months after her debulking surgery, the patient then gradually experienced recurring hypoglycemic episodes, with increasing intensity and frequency. She reported up to five hypoglycemic episodes per day, with blood glucose levels dropping down to as low as 30 mg/dL (1.7 mmol/L). Uninterrupted sleeping was no longer possible at this stage. The patient had to wake up at night to consume foods with a high glycemic index in order to artificially maintain blood glucose levels above 50 mg/dL (2.8 mmol/L).

**FIGURE 2:**
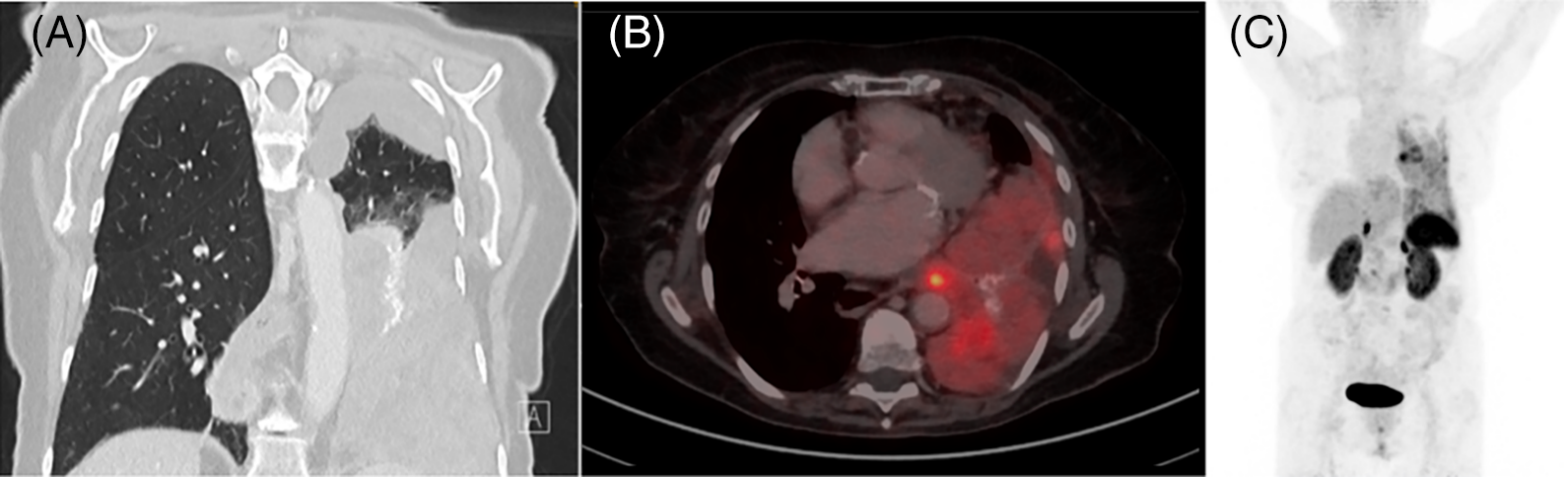
Imaging of the intrathoracic SFT before redebulking surgery. (A) Coronal chest‐CT showing a large heterogeneous tumor mass infiltrating the mediastinum, pleura, pericardium, and diaphragm. In addition, the expansion of the left lung is significantly reduced. (B, C) Transversal fusion (B) and maximum intensity projection (C) of [^68^Ga]DOTATATE/PET‐CT showing the SFT and further lesions in the pericardial fat tissue with variable and focally increased SSTR‐expression.

Radiological imaging showed significant tumor progression compared to the preceding 6 months. A total of 82 intrathoracic lesions were identified. Additionally, atelectasis of the left lower lobe was assessed. [^68^Ga]DOTATATE/PET‐CT revealed variable and focally increased SSTR‐expression of the SFT and further lesions in the pericardial fat tissue (Figure 2).

**FIGURE 3:**
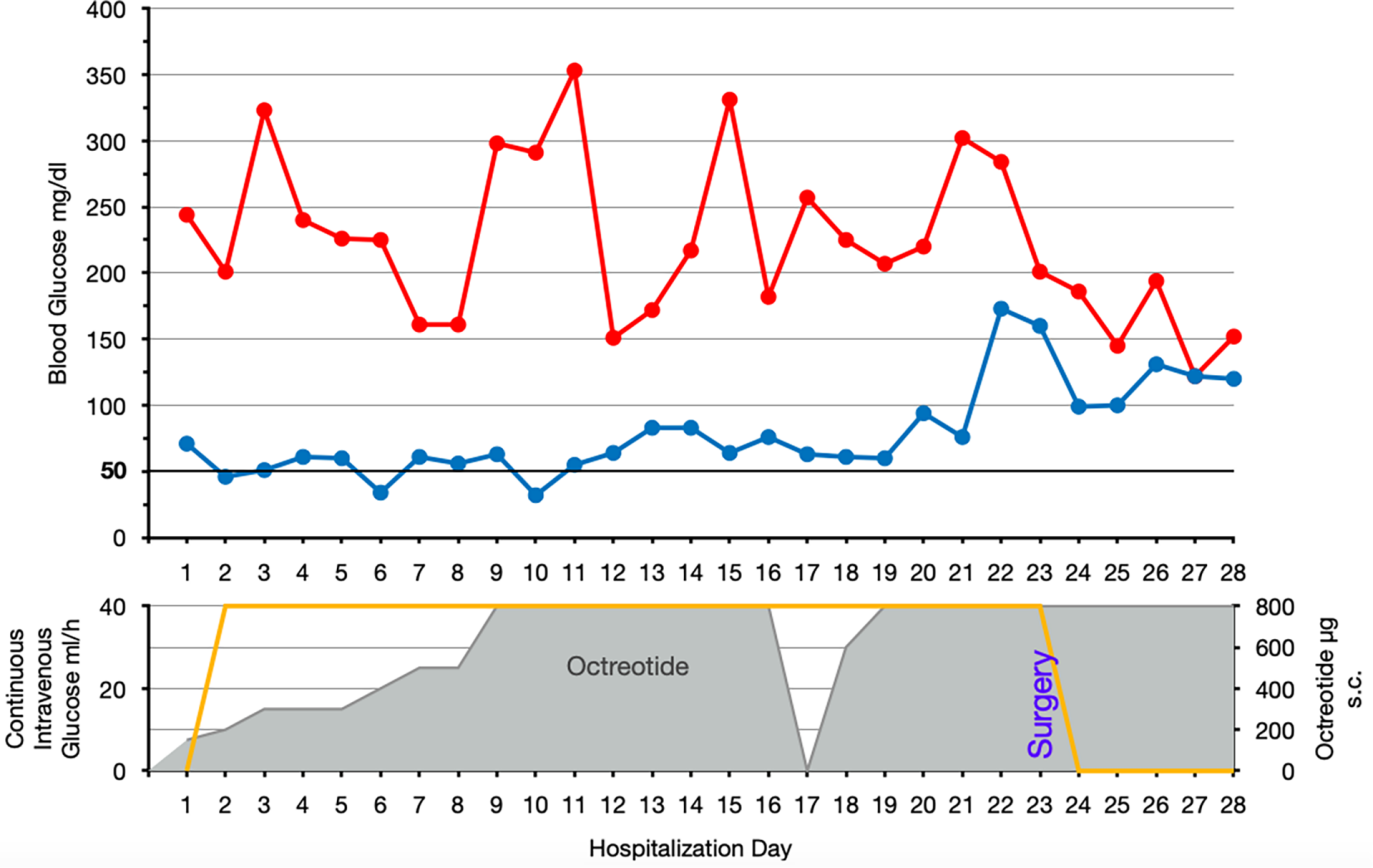
Blood glucose levels over the time course. A display of the patient's highest (red) and lowest (blue) blood glucose levels for each day of her hospital stay, along with the concurrent Octreotide (gray) and glucose therapy (orange). Octreotide was paused on day 17 because of a DOTA‐TATE‐PET/CT‐scan on day 18. The combination of 800 μg Octreotide per day and continuous intravenous glucose infusions maintained adequate blood glucose levels above 50 mg/dL. However, blood glucose levels were highly variable. Immediately after redebulking surgery on day 23, they stabilized at appropriate levels.

From the first day after her admission, the patient required continuous intravenous glucose infusions (intravenous glucose 20% with infusion rates of up to 40 mL/h) overnight, in order to prevent fatal hypoglycemic episodes. Since the tumor showed SSTR‐expressing lesions in the [^68^Ga]DOTATATE/PET‐CT, an adjuvant treatment with a somatostatin‐analogue, Octreotide, was started. While uptitration of Octreotide to a dose of 800 μg/day subcutaneously (4 × 200 μg) improved hypoglycemic episodes, continuous glucose infusions were still necessary to maintain tolerable blood glucose levels above 50 mg/dL (2.8 mmol/L) (Figure 3).

**FIGURE 4:**
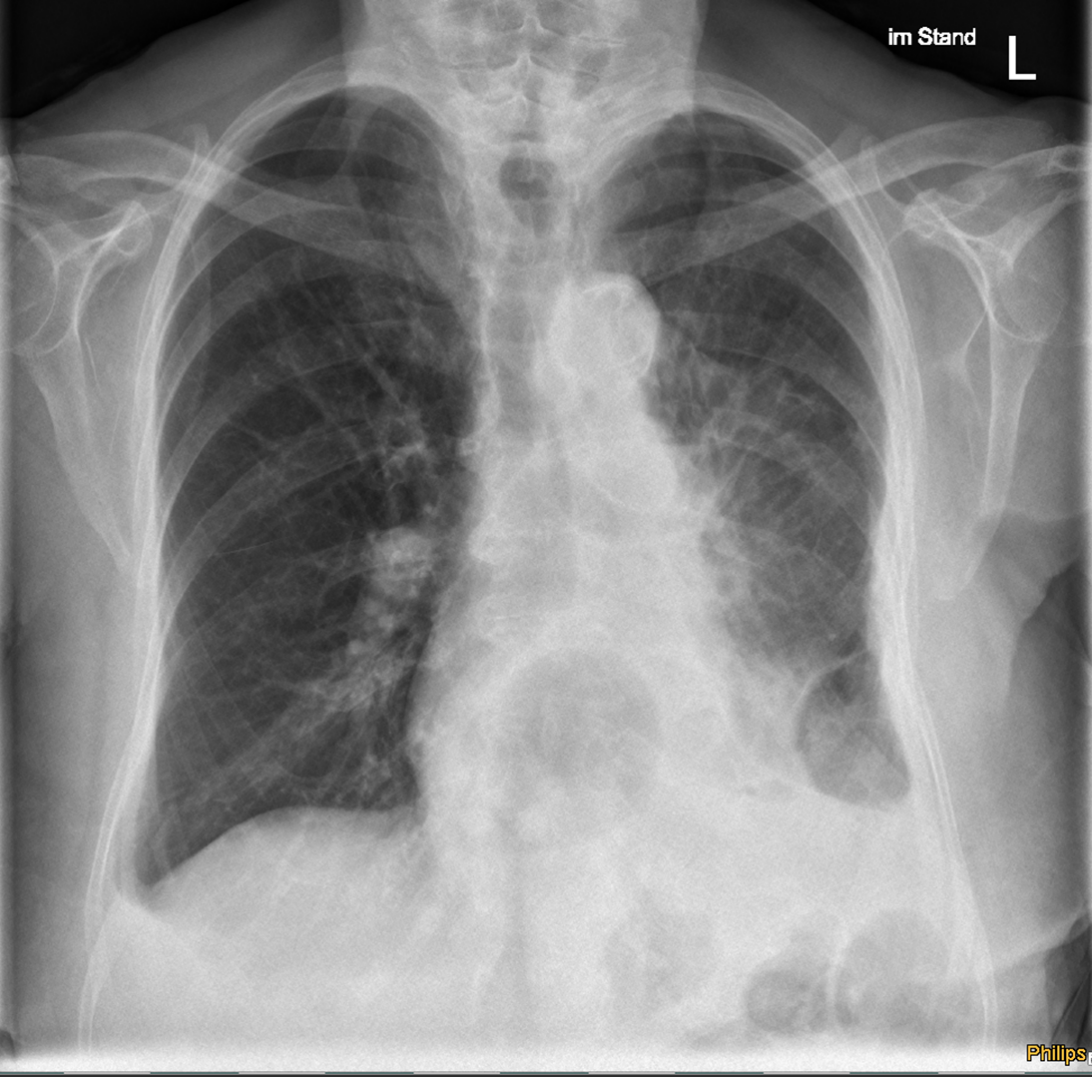
Chest radiograph of the intrathoracic SFT after redebulking surgery. Control chest‐x‐ray of the SFT after redebulking surgery showing a great reduction of tumor mass and improved expansion of the left lung.

**FIGURE 5:**
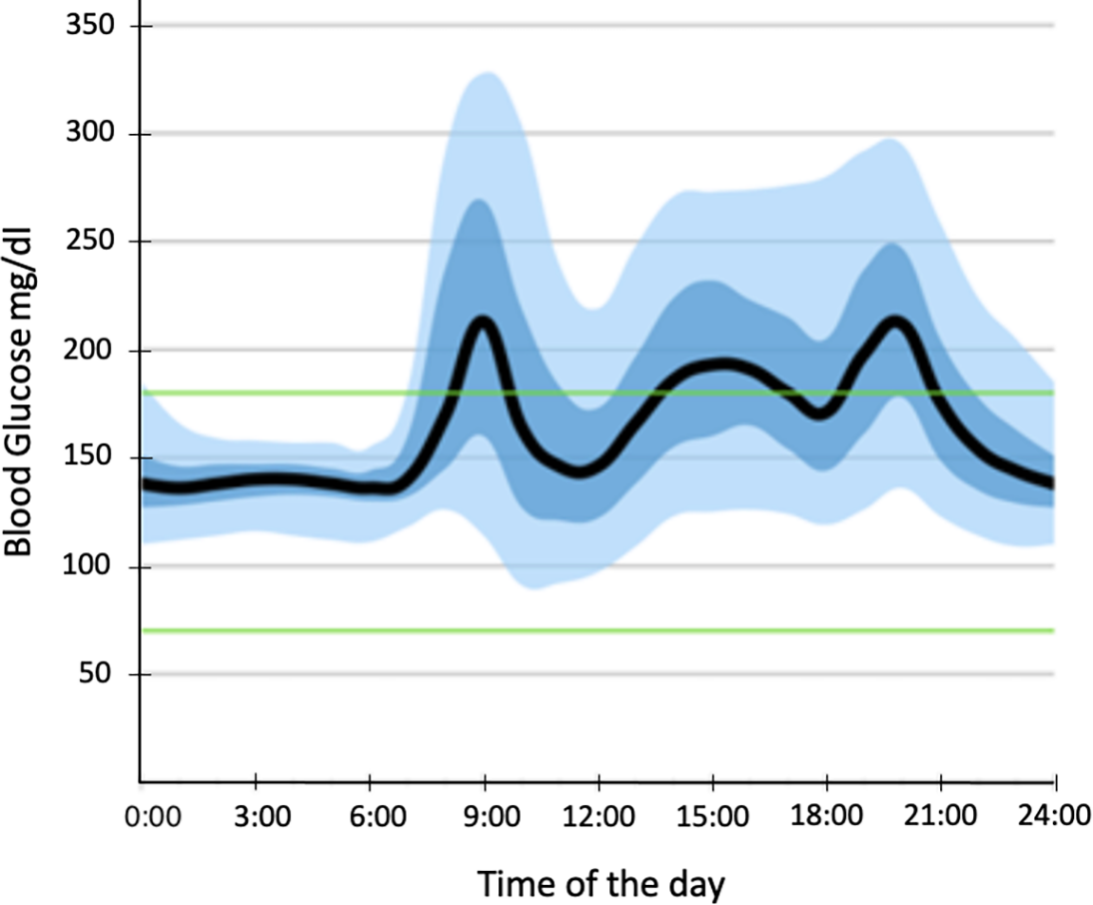
Daily blood glucose levels over the course of 4 months after redebulking surgery. Mean daily blood glucose levels (black) over the course of 4 months after redebulking surgery and under monthly treatment with Lanreotide. Interquartile‐range (dark blue) containing 50% and interdecile‐range (light blue) containing 80% of measured blood glucose levels. No more hypoglycemic episodes have been reported since redebulking surgery.

After weighing the potential age‐related risks and benefits, with informed consent of the patient, another debulking surgery was performed, removing great portions of the gelatinous, liquified tumor by suction and manual extirpation. Histological examination of the resected tissue again confirmed the diagnosis of the SFT, with fusiform neoplasia, elevated mitosis rates (5 per HPF) and without necrosis. Immunohistology showed that tumor cells were CD34(+), STAT6(+), EMA(−), CK1/3(−), and SMA(−), with a MIB‐1 determined proliferation rate of 15%. According to Demicco et al.,^22^ the tumor was classified as low risk. Glucose infusions could be terminated immediately after the surgery. Due to the inability to achieve R0 resection and considering the rapid tumor progression after her initial debulking surgery, the patient continued the Octreotide‐treatment aiming at a suppression of tumor proliferation and a reduction of hypoglycemic episodes in the future with now reduced tumor mass. Ten days after surgery, Octreotide was replaced with Lanreotide (120 mg/month subcutaneously) to allow a longer interval between injections. The patient was discharged after 32 days in our hospital. Continuous glucose monitoring (CGM) using a “Freestyle Libre 3” device was used to track the patient's blood glucose levels after her discharge. No hypoglycemic episodes have been reported since the redebulking surgery (Figures 4 and 5).

## 3 DISCUSSION

DPS is defined as hypoglycemia caused by paraneoplastic secretion of Big‐IGF‐II associated with intrathoracic SFT—a pleural neoplasm with an incidence of 0.2/100.000 per year.^23^ This condition is relatively rare, occurring in only 5% of patients with a SFT.^6^, ^7^ Locally recurrent DPS is particularly uncommon, reported in only 10% of cases.^3^ As their name implies, these tumors are usually solitary and fibrous. However, the patient in our case presented with multiple recurrent lesions of the lung, pleura and pericardial fat tissue. Gross tumor morphology had transformed into an atypical gelatinous, liquified tissue, with an increased proliferation rate of up to 15% underlying the early recurrence, which has not yet been described in the literature.

Early symptoms of hypoglycemia in patients with DPS include nervousness, sweating, tachycardia, and tremors, among others. In advanced stages, patients may develop headaches, confusion, syncopes, seizures, and central respiratory/circulatory disorders.^24^ Elevated IGF‐II levels may also lead to acromegaloid skin changes.^10^, ^11^ In November 2022, the advanced age of the patient combined with threatening hypoglycemic symptoms such as confusion and syncopes, required immediate symptomatic treatment.

Complete resection is considered the most promising treatment for patients with DPS.^10^, ^12^, ^17^ In this case, however, due to the unique tumor morphology and the pericardial involvement, an R0 resection was deemed unfeasible. The advanced age of the patient and her personal preferences rendered chemotherapy or radiotherapy untenable, as well. Instead, we started a symptomatic treatment with intravenous glucose infusions to bridge the time until a second debulking surgery. In many cases this has been reported as a sufficient strategy to prevent further hypoglycemic episodes.^3^, ^25^, ^26^

Although described as ineffective in current literature^20^, ^27^ an adjuvant treatment with Octreotide was initiated, because [^68^Ga]DOTATATE/PET‐CT scanning revealed SSTR‐expression of parts of the tumor lesions. Only in combination with Octreotide, intravenous glucose infusions successfully prevented fatal hypoglycemic episodes below 30 mg/dL (1.7 mmol/L) (Figure 3). These findings are consistent with a reduced intensity and frequency of hypoglycemic episodes in a 67‐year‐old patient with DPS treated with Octreotide.^21^ Due to the simultaneous application of glucose infusions and Octreotide, this single case report does not allow for any conclusions to be drawn about the individual effectiveness of both drugs. Therefore, it is difficult to quantify the distinct impact of Octreotide on the frequency of hypoglycemic episodes prior to the debulking surgery.

Somatostatin‐analogues bind to SSTR (preferentially type 2 and 5) and significantly inhibit proliferation and paraneoplastic secretion of SSTR‐expressing neuroendocrine tumors.^28^, ^29^ [^68^Ga]DOTATATE/PET‐CT allows for the visualization of SSTR‐2‐expression in tumors.^30^ Previous case reports described increased uptake in SSTR‐PET/CT in a SFT.^31^ To identify SSTR‐positive lesions, a [^68^Ga]DOTATATE/PET‐CT was performed prior to initiation of somatostatin‐analogue treatment. This scan revealed variable SSTR positivity in parts of the tumor mass (Figure 2). Therefore, it was not inconceivable that somatostatin‐analogue treatment could be potentially effective in reducing paraneoplastic secretion of IGF‐II‐derivatives and hypoglycemic tendency, and even slow down tumor progression in an SSTR expressing SFT, as well.

Due to the inability to achieve R0 resection and the rapid tumor progression after her first debulking surgery, the patient continued somatostatin‐analogue treatment after the second debulking surgery with the aim to potentially suppress rapid tumor proliferation and recurrence of hypoglycemia. Octreotide was later replaced by Lanreotide, a longer‐acting analogue of somatostatin in order to reduce the frequency of medical appointments and thus improve quality of life. Since then, no further hypoglycemic episodes have been reported (Figure 5). However, a period of 4 months may be too short in order to assess the benefit of somatostatin‐analogues on tumor proliferation in recurrent DPS. Especially since hypoglycemic episodes occurred as early as 1 year after the patients first debulking surgery in 2021.

As a single case report of a rare condition, it cannot reproduce the effects of somatostatin‐analogues on hypoglycemia in other patients with recurrent DPS and is limited regarding its generalizability. Therefore, the influence of somatostatin‐analogues on tumor progression of intrathoracic SFT remains elusive, and further research is needed to investigate their efficacy in recurrent DPS. However, the unique tumor‐morphology of the patient and the successful mitigation of symptoms using Octreotide in combination with intravenous glucose may help clinicians to deal with similar cases in the future.

We conclude that somatostatin‐analogues hold the potential of being effective in conjunction with limited surgical approaches for the treatment of hypoglycemia in recurrent or non‐totally resectable SFT entities underlying DPS. The CARE Guidelines where followed when preparing this case report ^32^ (Data S1).

## AUTHOR CONTRIBUTIONS

**Felix Schöler:** Conceptualization (lead); data curation (lead); formal analysis (equal); investigation (lead); visualization (lead); writing – original draft (lead); writing – review and editing (lead). **Maximilian Andreas Storz:** Conceptualization (equal); data curation (equal); formal analysis (equal); investigation (equal); resources (equal); writing – original draft (equal); writing – review and editing (equal). **Ashkan Khavaran:** Data curation (supporting); formal analysis (supporting); visualization (supporting); writing – original draft (supporting). **Nicolas Hümmler:** Data curation (equal); investigation (equal); resources (equal). **Maximilian Frederik Russe:** Data curation (equal); investigation (equal); resources (equal). **Christoph‐Ferdinand Wielenberg:** Data curation (equal); investigation (equal); resources (equal). **Katharina Laubner:** Data curation (equal); investigation (equal); resources (equal); supervision (equal). **Jochen Seufert:** Conceptualization (equal); data curation (equal); formal analysis (equal); project administration (lead); resources (equal); supervision (lead); writing – review and editing (equal).

## CONFLICT OF INTEREST STATEMENT

The authors have stated explicitly that there are no conflicts of interest in connection with this article.

## ETHICS STATEMENT

We declare that the work submitted to Cancer Reports has been done in accordance to “Wiley's Publication Ethics” guidelines and that is has been performed in an ethical and responsible way, with no research misconduct, which includes, but is not limited to data fabrication and falsification, plagiarism, image manipulation, unethical research, biased reporting, authorship abuse, redundant or duplicate publication, and undeclared conflicts of interest.

## CONSENT FOR PUBLICATION

Written and verbal informed consent were obtained from the patient prior to publication of her deidentified case report.

## Supporting information


**Data S1.** CARE checklist of information to include when writing a case report.

## Data Availability

Data sharing is not applicable to this article as no new data were created or analyzed in this study.
