# Supplementary material for: Effective management of recurrent Doege–Potter syndrome with somatostatin‐analogues: A case report
Source: Cancer Rep (Hoboken). 2024 Mar 5;7(3):e1992. doi: 10.1002/cnr2.1992 (PMC10913086; doi:10.1002/cnr2.1992)
Supplement: Supplementary file 1 — Data S1. CARE checklist of information to include when writing a case report. [file CNR2-7-e1992-s001.pdf]

CARE
case report guidelines

CARE Checklist of information to include when writing a case report

(cc) BY-NC-ND

Topic	Item	Checklist item description	Reported on Line
Title	1	The diagnosis or intervention of primary focus followed by the words "case report"	Line 3
Key Words	2	2 to 5 key words that identify diagnoses or interventions in this case report, including "case report"	Line 72 - 73
Abstract (no references)	3a	Introduction: What is unique about this case and what does it add to the scientific literature?	Line 51 - 65
	3b	Main symptoms and/or important clinical findings	Line 60 - 65
	3c	The main diagnoses, therapeutic interventions, and outcomes	Line 60 - 65
	3d	Conclusion—What is the main "take-away" lesson(s) from this case?	Line 67 - 69
Introduction	4	One or two paragraphs summarizing why this case is unique (**may include references**)	Line 111 - 114
Patient Information	5a	De-identified patient specific information.	Line 116 - 119
	5b	Primary concerns and symptoms of the patient.	Line 118 - 132 + 156 - 161
	5c	Medical, family, and psycho-social history including relevant genetic information	Line 130 - 132
	5d	Relevant past interventions with outcomes	Line 128 - 153
Clinical Findings	6	Describe significant physical examination (PE) and important clinical findings.	Line 130 - 132 + 142 -143
Timeline	7	Historical and current information from this episode of care organized as a timeline	Line 128 - 194
Diagnostic Assessment	8a	Diagnostic testing (such as PE, laboratory testing, imaging, surveys).	Line 133 - 137 + 143 - 153 + 158 - 176 + 181 -185
	8b	Diagnostic challenges (such as access to testing, financial, or cultural)	did not apply in this case
	8c	Diagnosis (including other diagnoses considered)	Line 134 - 135
	8d	Prognosis (such as staging in oncology) where applicable	Line 186 - 189 ???
Therapeutic Intervention	9a	Types of therapeutic intervention (such as pharmacologic, surgical, preventive, self-care)	Line 135 - 136 + 146 - 147 + 172 - 178 + 180 - 191
	9b	Administration of therapeutic intervention (such as dosage, strength, duration)	Line 172 - 178 + 189 - 190
	9c	Changes in therapeutic intervention (with rationale)	did not apply in this case
Follow-up and Outcomes	10a	Clinician and patient-assessed outcomes (if available)	Line 192 - 194
	10b	Important follow-up diagnostic and other test results	Line 192 - 194
	10c	Intervention adherence and tolerability (How was this assessed?)	did not apply in this case
	10d	Adverse and unanticipated events	did not apply in this case
Discussion	11a	A scientific discussion of the strengths AND limitations associated with this case report	Line 235 - 248 + 260 - 267 + 268 - 274
	11b	Discussion of the relevant medical literature **with references**.	Line 217 - 277
	11c	The scientific rationale for any conclusions (including assessment of possible causes)	Line 235 - 274
	11d	The primary "take-away" lessons of this case report (without references) in a one paragraph conclusion	Line 275 - 277
Patient Perspective	12	The patient should share their perspective in one to two paragraphs on the treatment(s) they received	did not apply in this case
Informed Consent	13	Did the patient give informed consent? Please provide if requested	Yes ☒ No ☐
